# Functional Selection of shRNA Loops from Randomized Retroviral Libraries

**DOI:** 10.1371/journal.pone.0043095

**Published:** 2012-08-17

**Authors:** Stig Mølgaard Rask Jensen, Alexander Schmitz, Finn Skou Pedersen, Jørgen Kjems, Jesper Bertram Bramsen

**Affiliations:** 1 Department of Molecular Biology and Genetics, Aarhus University, Aarhus, Denmark; 2 Interdisciplinary Nanoscience Center (iNANO), Aarhus University, Aarhus, Denmark; Beckman Research Institute of the City of Hope, United States of America

## Abstract

Gene silencing by RNA interference (RNAi) can be achieved by the ectopic expression of tailored short hairpin RNAs (shRNAs) which after export to the cytoplasm are processed by Dicer and incorporated into the RNA induced silencing complex (RISC). Design rules for shRNAs have been the focus of several studies, but only a few reports have turned the attention to the sequence of the loop-region. In this work we selected high-functional and low-functional shRNA loops from retroviral hairpin-loop-libraries in an RNAi reporter assay. The procedure revealed a very significant and stem sequence-dependent effect of the loop on shRNA function and although neither strong consensus loop sequence nor structural motifs could be identified, a preferred loop sequence (5′-UGUGCUU-3′) was found to support robust knock down with little stem sequence dependency. These findings will serve as a guide for designing shRNAs with improved knock down capacity.

## Introduction

The phenomenon of RNAi in mammals is usually initiated through either the production of microRNAs (miRNAs), which control endogenous mRNA stability or translation levels [1], [2], [3] or by the production of small interfering RNAs (siRNAs) from double stranded RNA (dsRNA) of either exogenous or endogenous origin [4], [5]. miRNAs are transcribed as primary miRNA transcripts (pri-miRNA), which are co-transcriptionally cleaved into precursor miRNAs (pre-miRNAs) by the microprocessor complex comprised of DGCR8 and the RNaseIII enzyme, Drosha. The pre-miRNAs are small irregular hairpin structures that are exported by the Exportin5/RanGTP complex to the cytoplasm where they are recognised and processed by the RNaseIII enzyme Dicer in conjunction with TRBP, PACT, and Ago2, reviewed by Rana *et al.* [6]. The result is irregular double stranded RNA, of which only one strand, the miRNA, is retained after transfer to the RNA induced silencing complex (RISC) [7], [8], [9]. The activated RISC recognises cognate mRNAs through base pairing with the miRNA leading to mRNA destabilization, translational inhibition or mRNA cleavage [6].

The siRNAs are naturally either generated from long exogenous dsRNAs such as viral RNAs or from endogenous convergent transcripts from overlapping genes [5]. Dicer cleaves these dsRNAs into short ∼21 bp siRNAs which enter RISC and induce degradation of their innate origin RNA (reviewed in [6]).

A common strategy in knockdown experiments is to combine features from both the miRNA and the siRNA pathways and express short hairpin structures (i.e. shRNAs) from Pol III promoters. This bypasses the microprocessor processing step and usually provides a strong and stable intracellular siRNA production. Numerous vectors, of both viral and non-viral origin, have been produced for expression of various shRNA cassettes, but few studies have attended to the optimization of the loop sequences for improved function. It has been suggested, based on comparison of miRNA-loops, that the loop sequences only provide marginal effect on RNAi efficacy [10] whereas other studies show clear differences [11], [12], [13], [14]. In the most elaborate study on the functionality of loop structure it was recently reported that the loop sequence is decisive for shRNA functionality, and that clear structural preferences exist [14]. To explore the structural preferences in more depth, we took advantage of a cell based retroviral selection assay and screened a large number of different loop sequences of three different sizes for low-functional and high-functional sequences. Our analysis reveals that shRNA efficiency is indeed very loop-dependent, that loops affect shRNA processing by RNAi proteins and that shRNA loop efficacy can be stem-sequence dependent. We also propose an optimal shRNA loop for general use in shRNA design.

## Results

### Selection of functional loop sequences

**Figure 1:**
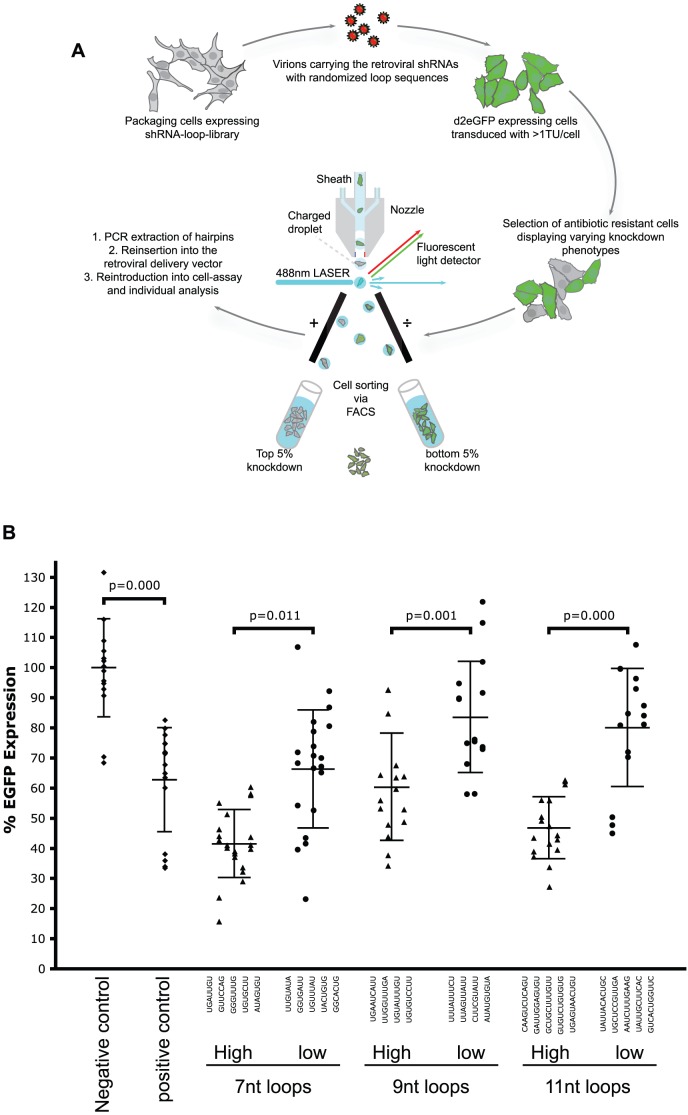
The shRNA loop co-determine shRNA efficacy. **A**. Schematic representation of the shRNA-loop selection experiment. DNA fragments encoding shRNA hairpins with randomised loop sequences of 7, 9, or 11 nucleotides in length were inserted into a retroviral expression cassette and packaged into viral particles using a packaging cell line. Infection of eGFP-expressing cells at low ratio of infectious units to the number of cells insured clonal expression of individual shRNA hairpins from genomically integrated retroviral vectors. Cells sorted according to eGFP expression level by FACS to isolate cell pools expressing high- and low-functional shRNA, respectively. The loop sequences were amplified from the two pools and recloned into the retroviral vector that subsequently was packaged and reintroduced into eGFP expressing target cells. **B**. Histogram showing the eGFP expression levels obtained after re-introducing randomly selected clones from functional pool of retroviral vectors. *Indicates a positive control loop sequence previously published by Brummelkamp et al. [11].

To screen for RNAi competent loop sequences in shRNA, three libraries with a constant 19 base pair (bp) stem sequence directed towards a validated eGFP target [15] and a random 7-, 9-, or 11-nucleotide loop (shRNA-7, shRNA-9 and shRNA-11) were constructed and cloned into a retroviral vector behind a H1-promoter. The plasmids were packaged in the packaging cell-line, PLAT-E [16]), and tranduced into HeLa cells stably expressing a destabilised version of the eGFP (with a half life of 2 hours) and the ecotropic receptor of MoMLV. To favor clonal expression of individual vector constructs, transducing unites were kept at ∼0.03–0.17 TU/cell. Untransduced cells were removed by puromycine-selection and the remaining cells were analysed for eGFP expression by flow cytometry. From each of the three cell libraries, 5% of the cells that either displayed the highest or lowest eGFP expression were sorted using fluorescence activated cell sorting (FACS; data not shown; cf. figure 1A for an overview of the experimental procedure). To confirm that the observed differences in shRNA efficacy were not merely integration site dependent effects affecting shRNA expression levels, selected hairpin-constructs from each of the six selected pools were retrieved by PCR, reintroduced into the retroviral expression vector, re-transduced into the reporter cell line and individual cell clones were analysed by flow cytometry. Overall, we found that the hairpins from the three selected library-pools, that displayed the highest degree of knockdown in the first selection round, remained the most potent when re-introduced and tested individually. The potency was moreover largely independent of the site of integration. Similarly, the least functional shRNA pools remained functionally inefficient after re-transduction (figure 1B). This shows that the shRNA loop has an important influence on shRNA function and that our selection strategy can indeed distinguish differences in loop efficiency.

### Defining the structure of a functional loop

**Figure 2:**
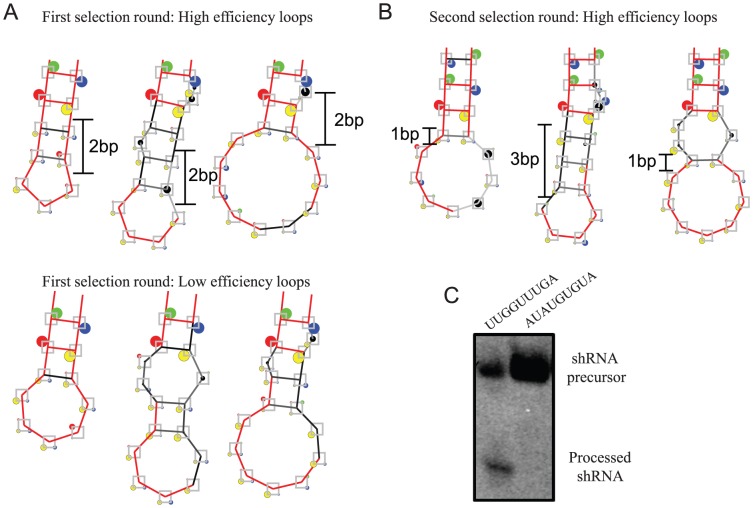
*In silico* analysis of the sequences from the loop-selections. Consensuses secondary structures of high and low functionality loops predicted by RNAforester [20] based on MFold3.2 generated structures after the 1^st^ selection round (**A**) and 2^nd^ selection round (**B**). Loops of 7 nt, 9 nt and 11 nt loop libraries are shown from left to right. The figure shown uses standard RNAforester output settings: Each base position is represented by a square where the corners represent the four bases with a dot. The size of the dot represents the frequency of the particular base; colour code: Red-A, yellow-U, green-C, blue-G, black circle: the frequency of a gap is proportional to a black circle growing at the centre of the square. Bases or base pair bonds that have a frequency of one hundred percent are drawn in red color. The blue arrow indicates the last base pair of the duplex stem region. Sequences displaying a stretch of 4 or more uracils, have been removed to avoid contribution from transcripts terminating prematurely [18], [19]. (**C**). Evaluating shRNA processing by northern blotting. shRNA RNA vectors harboring shRNA targeting sequence #1 and the indicated loops were transiently transfected into H1299 cells and shRNA processing were evaluated by 15% denaturing PAGE and northern blotting using a 19-mer probe against the processed eGFP antisense strand of the shRNA. Both the mature 51-nt and processed 21-nt RNA species are identified for the efficient loop where no processing is seem for the inefficient loop.

Having validated our assay we next analysed differences in the loops composition between high-functional and low-functional shRNA by sequencing shRNA expression cassettes from the all six pools from the first selection round (data not shown). No alterations in the promoter nor shRNA stem regions were detected confirming that differences in knockdown efficiencies were caused by the loop (and to some extend integration site). The presence of a potential loop sequence-motif in the individual libraries was assessed using weblogos [17], however, no apparent sequence motif appeared to be selected in any of the libraries. To search for a potential secondary-structural motifs, the loops were examined in MFold 3.2 [18], [19] following a structural comparison using the RNAforester1.5 program [20]. This study suggested that the least RNAi competent loops exhibit more intensive base pairing throughout the loop, an effect that seemed most easily identifiable for the shortest 7-mer loops that hold less potential to fold into complex tertiary structures as compared to the longer 11-mer loops (figure 2A). In contrast, the consensus structures from the pool exhibiting the highest RNAi efficiency, all display less base pairing in the loop-extremity but a 2-bp extension of the stem. Hence, the 19-bp hairpin stem that was used in all libraries seemed to be extended to 21-bp stems in efficient shRNAs which apparently increases the RNAi functionality, likely by affecting shRNA processing by RNAi proteins. To test this, we transiently transfected vectors expressing shRNA containing either the highly functional 9-mer loop 5′- UUGGUUUGA-3′ or the inefficient loop 5′-AUAUGUGUA-3 into H1299 cells and evaluated shRNA processing by northern blotting. Indeed, we found that the efficient shRNA was processed by Dicer into mature single stranded ∼21-mer RNAs whereas the nonfunctional shRNA was not processed leading to a remarkable buildup in shRNA levels (figure 2C). This suggest that difference in shRNA loops performances may well be attributable to differences in shRNA processing by Dicer which is in agreement with the observation of a preference for a 2-bp extension, although we cannot exclude differences in nuclear export by the Xpo5-complex. To increase the stringency of the selection, the pools containing the superior 5% loop sequences were re-introduced into the same retroviral vector and reselected as described above. Again, the 5% of the cell populations that displayed the highest degree of knockdown were isolated via FACS and the hairpin-constructs were retrieved and sequenced. Weblogos were again used to display potential sequence motifs (data not shown) and MFold3.2/RNAforester1.5 analysis was applied to examine for secondary structural motifs (figure 2B). Still no apparent sequence motif arose in any of the libraries. However, in accordance with the structural consensuses obtained from the first selection round the RNAforester analysis show little loop-structure, except from a 1–2 base pair extension of the stem. The minor differences in the secondary consensus structure predictions between the 1^st^ and 2^nd^ selection rounds may, however, be a consequence of tertiary structure selection involving non-canonical-Watson-Crick base pairing [21], [22], [23] that are not predictable by the analysis applied. Overall, these data still suggest that RNAi-competent loops should be structurally compatible with efficient Dicer processing rather than relying on specific primary sequence preference.

### Loop dependencies on stem-sequence

**Figure 3:**
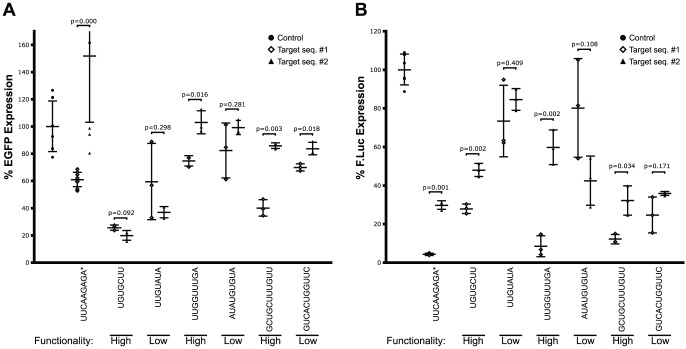
Testing loops in context of different stem-sequences. The shRNA cassettes were either introduced stably using retroviral vector (**A**) or transiently from a plasmid co-transfections with a firefly luciferase reporter construct containing the eGFP target sequence (**B**). eGFP levels were measured by Flow cytometry. Asterisk denotes the control shRNA containing the loop previously published by Brummelkamp et al. [11].

The efficacy of shRNA loops may depend on the sequences of the double stranded segment in the shRNA. Therefore, new shRNA constructs were synthesised targeting another eGFP sequence yet containing the most and least favourable loops from our selection. The knockdown efficacy of these constructs were tested in two reporter-assays: by stable expression using the retroviral system described above and transiently from a plasmid where the cognate eGFP target sequence was inserted into the 3′ UTR of the firefly luciferase gene (*luc*), proximal to the *luc*-ORF. Simultaneous expression of *Renilla* luciferase was used as a transfection control in the H1299 cells employed in the assay. Substitution of the stem had pronounced negative effect on the knockdown efficacy for most efficient loops which likely reflects that the target sequence #2 is less efficient than the first siRNA sequence *per see*. Still, the drop in silencing activity was particularly pronounced for some loop sequences (e.g. figure 3A loop UUGGUUUGA) and some poorly performing loops even exhibited better activity with the second target sequence (e.g. figure 3A loop UUGUAUA and figure 3B loop AUAUGUGUA), which suggest that the influence of the loop can be stem-dependent. Notably, we identify a shRNA loop sequences (e.g. UGUGCUU) that support highly efficient RNAi in a seemingly stem sequence independent manner upon stable integration (figure 3B, loop UGUGCUU) and we hereby recommend this as a potentially universal loop in shRNA designs.

## Discussion

In the present study the impact of the loop sequence of shRNAs on RNAi efficacy was investigated using shRNA libraries with randomized loop nucleotide composition. The data show that the loop sequence plays a significant role in shRNA efficacy likely by influencing its nuclear export by Xpo5 or subsequent processing by Dicer in the cytoplasm [24], [25], [26]. Today, there is no evidence that the loop participates in Xpo5 binding which is rather mediated by interactions with the stem and 3′ overhang [27]. Therefore, shRNA loops are more likely to influence the shRNA processing by the RNAi machinery in the cytoplasm and RNA hairpins are indeed described as loop-sequence-specific substrates of double stranded RNA binding domains (dsRBDs) [28]. A most likely candidate for differential shRNA recognition is the Dicer-dsRBD as the dsRBDs in other cytoplasmic proteins TRBP and PACT seem rather implicated in protein-protein interactions [29], [30], [31]. Also, from the data presented here, all the consensus structures from the pool, exhibiting the highest RNAi efficiency, display less base pairing in the loop-extremity but a 2-bp extension of the 19-bp fixed stem, which is compatible with a model where the Dicer-dsRBD is responsible for substrate specificity. In agreement, shRNAs with longer 25–29-bp stems are less loop-dependent as compared to 19-bp shRNAs [32], [33]. The presented data also suggest that putative optimal loop motifs are not directly based on a readily distinguishable nucleotide sequence. This is in good correspondence with the solved structure of a *Saccharomyces Cerevisiae* RNaseIII (Rnt1p) dsRBD-tetraloop complex, which shows that protein interactions occur via the RNA sugar backbone and not via the nucleotide bases in the hairpin loop. In fact, the bases from the top of the stem and the loop bases form a twisted stack via non-Watson-Crick interactions to form a specific tertiary motif that specifies it as a dsRBD substrate [34]. Hereby the existence of a tertiary loop-motif or signature in the shRNA loop may similarly direct the function of the Dicer-complex machinery to influence the RNAi efficacy and explain the result presented here. In this regard, the size of the loop itself could vary with several nucleotides without affecting the recognition ability as long as a certain plasticity exists within the loop-nucleotides that allow optimal tertiary structures to be formed. Another recent study demonstrated a clear loop effect on shRNA functionality [35]. In this study, the various base-requirements were addressed by mutational analysis of a limited number of shRNA loops, rather than selection-procedures presented here which comprises all possible loop sequences. Corroborating with the findings of the present study, it was shown that 19-bp stem-loops harbour a preference, although not absolutely, for loops that may extend the stem by approximately 2-bp, and that the size of the loop is not the definitive factor. Notably the data presented here show that loop sequences optimised for one stem sequence may not function in the context of another. However, some loops appear to be less dependent on stem-sequences, and can, at least in context of the stems tested here, be regarded as stem-independent. An example from this work is the UGUGCUU loop, which may successfully be utilized as a prime loop candidate in future RNAi experiments.

## Materials and Methods

### Oligos and vectors

 The retroviral libraries were made from pSUPER-retro-pur™ (OligoEngine™), that drives expression of the shRNA from an H1 promoter. The shRNA expression cassettes were generated from oligoes with stretches of 7, 9, or 11 random nucleotides: 5′-GACGGGATCCCCGGCTACGTCCAGGAGCGCA-N_7, 9, 11_-TGCGCTCCTGGACGTAGCCTTTTTGGAAAAGCTTACGA-3′, where the subscript N_7, 9, 11_ denotes the randomized region. The stem region is targeted to a previously validated eGFP mRNA sequence [15]. The single stranded DNA oligo-libraries (DNA Technology Aps) were transformed into double stranded fragments by primer-extension using the following primer: 5′-TCGTAAGCTTTTCCAAAAAGGCTACGTCCAGGAGCGCA-3′. To avoid that the single stranded library-oligoes form hairpins during the extension reaction, short oligoes (5′-ACGTAGCCGGGGATCCCGTC-3′, 5′-GTAGCCGGGGATCCCGTC-3′and 5′-AGCCGGGGATCCCGTC-3′) were added in excess. This strategy facilitated construct formation via dynamic base exchange [36]. DNA libraries were purified on a agarose gel and extracted using either spin-columns (Millipore, cat.no.: LSKGEL050) or by electroelution (in an apparatus equivalent to the Extrophor; Pharmacia-LKB). Poly-acrylamid gel purifications were done either via Ultrafree-MC spin-columns (Millipore) or via electroelution.

#### Cell cultures

HeLa, HeLa-d2eGFP-mCAT, PLAT-E [16], and H1299 cells were cultured in Dulbecco's modified eagle's medium, (D-MEM) supplemented with 10% fetal bovine serum (FBS) and 1% Penicillin/Streptomycin (Pen/Strep). Transfections were performed either with CaPO_4_ co-precipitation [37], HeLa-MONSTER (Invitrogen) or with lipofectamine (Invitrogen) in accordance with the manufactures recommendations. Transductions were done after sterile filtration (0.2 µm: Sartorius, Minisart: Vivascience), and applying 6 mg/ml Polybrene® (Sigma-Aldrich) to facilitate membrane fusion. Antibiotic selections were carried out 48 hours post-transductionally with 1,5 µg/ml Puromycine (Sigma-Aldrich). As transfection control pDSred1-N1 (Clontech) was co-transfected allowing visual transfection validations. Stable cell lines expressing mCAT were generated by transfecting pmCAT IR HYG MSS (Pharmexa) into HeLa cells, following a long term selection with 100 µg/ml Hygromycin B (Invitrogen).

### Flow cytometry

For flow-cytometry cells were thoroughly treated with Trypsin+EDTA, centrifuged at 12000 rpm (∼800 g) and re-suspended in phosphate buffered saline (PBS) supplemented with 20% FBS. Then stored briefly @ 4°C until run on the flow-cytometer. Cell preparations for sorting via FACS were re-suspended in Hank's buffered salt solution (HBSS, Gibco) supplemented with 20% FBS, and further filtered in 5 ml Falcon filter tubes (BD bioscience) prior to usage. Genomic DNA was purified through usage of DNAzol® (Molecular Research Center, Inc.), in accordance with the manufactures protocol. Flow cytometry was done either on a FACS-calibur or on a FACS-vantage SE (for sorting) both with standard lasers and detectors (BD bioscience). For data acquisition and analysis Cell Quest Pro (BD) was employed.

### PCR and Sequencing

PCRs were run either with Hi-fidelity (Roche) of Pfu-polymerases with or without 10%, DMSO (Invitrogen). PCR fragments were cloned for sequencing via TOPO® cloning (Gateway® Technology, Invitrogen™). Sequencing was done with BigDye3.1 (Applied Biosystems) chemistries supplemented with either 10% DMSO (Invitrogen) or with 0.83 M Betaine (Sigma-Aldrich) and 1× PCRx Enhancer (Invitrogen) to increase the hairpin-sequence read-through [38], otherwise in accordance with the manufacturers protocol.

PCR-primers: pSUPER-retro-Fwd: 5′-CTCACTCCTTCTCTAGGCGCCGGAATTAGA-3′; pSUPER-retro-Rev: 5′-ACGGAGCCGGTTGGCGCCTACCGGTGGATG-3′

Sequencing primers: FWD: 5′-GCCGGAATTAGATCGATCTC-3′; Rev: 5′-CGAACGCTGACGTCATC-3′

#### Evaluation of shRNA processing by Northern blotting

H1299 cells were transfected in 6 well format by 1 microgram of the indicated shRNA expression vector using Lipofectamine (Invitrogen) and total RNA was harvested after 48 hours using Trizol reagent (Invitrogen). Twenty micrograms of RNA was precipitated and resuspended in 1× Urea loading buffer (0.05% (w/v) Bromophenol Blue,8 M Urea, 0.05% (w/v) Xylene Cyanol, 1 mM EDTA, 20 mM Tris, pH 8.0) and denatured at 95°C for 1 min. and directly loaded onto a 15% polyacrylamide/8 M urea (SequaGel, National Diagnostics) and run at 12 watts for ∼1 hour. Separated RNA was electro-blotted onto a Hybond-N+ membrane (Amersham). After UVcross-linking and air-drying, blotted membrane were pre-hybridized in Church buffer (1 mm EDTA, 0.5 m Na2HPO4, pH 7.2, and 7% SDS) at 37°C for ∼3–4 hours and hybridized overnight with a ^32^P-end-labeled DNA probe (sequence: 5′-GGCTACGTCCAGGAGCGCA-3′). The membrane was washed 2–4 times at RT with 2× SSC and 0.1% SDS exposed by phosphor-imaging using a Typhoon Scanner System.

#### Luciferase assay

To measure knockdown via luciferase, eGFP target sequences were inserted into the *Sac*-I- and *Nhe*-I-sites of pISO downstream from the *luc*-ORF.Primers: 1^st^ target: Sense strand (fwd): 5′-CGGCTACGTCCAGGAGCGCACCG-3′; anti-sense strand (rev): 5′-CTAGCGGTGCGCTCCTGGACGTAGCCGAGCT-3′; 2^nd^ target: Sense strand (fwd): 5′-CACGACGTAAACGGCCACAAGTTCGAATTCG-3′; anti-sense strand (rev): 5′-CTAGCGAATTCGAACTTGTGGCCGTTTACGTCGTGAGCT-3′. The assay was conducted by co-transfecting the target-site pISO plasmid, a *Renilla*-Luciferase plasmid as an internal transfection control, and either of the selected pSUPER-retro-sh-loop constructs. 48 h post-transfection, cells were lysed and luciferase intensities measured using a Dual-Luciferase kit (Promega) and a luminometer (Lumat LB 9501, Berthold).
